# Fine-Mapping Angiotensin-Converting Enzyme Gene: Separate QTLs Identified for Hypertension and for ACE Activity

**DOI:** 10.1371/journal.pone.0056119

**Published:** 2013-03-04

**Authors:** Chia-Min Chung, Ruey-Yun Wang, Cathy S. J. Fann, Jaw-Wen Chen, Yuh-Shiun Jong, Yuh-Shan Jou, Hsin-Chou Yang, Chih-Sen Kang, Chien-Chung Chen, Huan-Cheng Chang, Wen-Harn Pan

**Affiliations:** 1 Institute of Biomedical Sciences, Academia Sinica, Taipei, Taiwan; 2 Division of Preventive Medicine and Health Service Research, National Health Research Institutes, Miaoli, Taiwan; 3 Department of Public Health, China Medical University, Taichung, Taiwan; 4 Cardiovascular Research Center, National Yang-Ming University, Taipei, Taiwan; 5 Department of Medical Research and Education, Taipei Veterans General Hospital, Taipei, Taiwan; 6 Department of Cardiology, Tao-Yuan General Hospital Department of Health, Tao-Yuan, Taiwan; 7 Institute of Statistical Science, Academia Sinica, Taipei, Taiwan; 8 Department of Cardiology, Min-Sheng Hospital, Taoyuan, Taiwan; 9 Department of Cardiology, Lin-Shin Hospital, Chungli, Taiwan; Tulane School of Public Health and Tropical Medicine, United States of America

## Abstract

Angiotensin-converting enzyme (ACE) has been implicated in multiple biological system, particularly cardiovascular diseases. However, findings associating *ACE* insertion/deletion polymorphism with hypertension or other related traits are inconsistent. Therefore, in a two-stage approach, we aimed to fine-map *ACE* in order to narrow-down the function-specific locations. We genotyped 31 single nucleotide polymorphisms (SNPs) of ACE from 1168 individuals from 305 young-onset (age ≤40) hypertension pedigrees, and found four linkage disequilibrium (LD) blocks. A tag-SNP, rs1800764 on LD block 2, upstream of and near the ACE promoter, was significantly associated with young-onset hypertension (p = 0.04). Tag-SNPs on all LD blocks were significantly associated with ACE activity (p-value: 10^–16^ to <10^–33^). The two regions most associated with ACE activity were found between exon13 and intron18 and between intron 20 and 3′UTR, as revealed by measured haplotype analysis. These two major QTLs of ACE activity and the moderate effect variant upstream of ACE promoter for young-onset hypertension were replicated by another independent association study with 842 subjects.

## Introduction

Angiotensin I converting enzyme (ACE, EC 3.4.15.1) is a key protein in the renin-angiotensin-system (RAS) that cleaves angiotensin I to form angiotensin II and plays important roles in sodium homeostasis and blood pressure control. However, ACE-targeted therapeutic effects exceed what is attributable to blood pressure reduction, including actions against platelet adhesion and aggregation, inflammation, trophic effects, and atherosclerosis [1]. In addition, *ACE* polymorphisms have been associated with problems such as cardiovascular diseases, diabetic nephropathy [2], renal failure, [3] pulmonary sarcoidosis, [4] and Alzheimer's disease [5]. We also found that a SNP on exon17 was associated with not only ACE activity but also blood pressure response to ACE inhibitors (ACEI). If we can further pinpoint the critical region influencing ACE activity, the information may contribute to personalized medicine.

The variation of this gene and its genetic impact on diseases among different populations are not well understood, although *ACE* has been extensively researched. Because ACE activity has a reasonably high heritability with estimates ranging from 0.2 to 0.7 [6], [7] and represents an upstream and internal facet of hypertension; it is therefore considered a good endophenotype, by the criteria outlined in Pan et al. [8], for discovering variants with potential for involvement in blood pressure regulation, cardiovascular function, and other biological processes.

Strong evidence exists for an association between the ACE Alu insertion/deletion allele (I/D) found in intron 16 and plasma ACE activity, with increased levels among people with the “deletion” allele [6], [9]. An immense number of studies published thus far have investigated the association between the I/D polymorphism and numerous clinical outcomes. However, the results concerning the association have been conflicting [9], [10]. The I/D polymorphism does not have a known functional effect and is considered a surrogate marker in linkage disequilibrium (LD) with a functional variant directly affecting plasma ACE level. It is proposed that other candidate polymorphisms within this gene may contribute to the mechanism affecting plasma ACE activity and the putative health sequelae.

Because of the high degree of LD between the polymorphisms within the ACE gene, it is difficult to distinguish functional variants from neutral polymorphisms occurring on the same haplotypes. Several studies have applied a variety of methods in various populations to narrow down the location of quantitative trait loci (QTL) of ACE activity on *ACE*. Despite considerable effort, the precise location of the functional polymorphism is still undetermined. Summarizing findings from previous studies, it is most likely located in either of two regions: the first spanning from upstream, promoter to the 5′ breakpoint, and the other between intron 18 and the 3′UTR [7], [11], [12], [13], [14], [15], [16].

Previous studies have been limited by relatively small samples and a limited number of markers on the *ACE* gene. Our experimental strategy is to adopt a high-resolution genetic mapping scheme of the ACE gene using a reasonably-sized sample of Han Chinese who have genetic differences from the Caucasian population.

Fine-mapping potential QTLs for ACE activity has not been reported in Han Chinese. Therefore, following up on our previous genomewide scan study [17] we set out to map QTLs for serum ACE activity and to examine their effects on hypertension by analysis of variance approach and measured haplotype analysis. We employed a two-stage approach using, first, data from a Taiwan young-onset hypertension family study and, second, information from an Academia Sinica multi-center study of young-onset hypertension.

## Methods

### Ethics statement

This study was approved by the Human Investigation Committee of the Institute of Biomedical Sciences, Academia Sinica. Written informed consent was obtained from every participant during his/her initial clinic visit.

### Subjects

Data from a study of young-onset hypertension families in Taiwan was used to find QTLs for ACE activity and the locus for hypertension. Data from a young-onset hypertension multi-center study conducted by Academia Sinica was used to replicate the findings.

### Young-onset hypertension family study and phenotype data

The study was carried out with data collected from 1168 individuals (597 men and 571 women) from 305 hypertension pedigrees. The hypertension probands for this study came from the Chu-Tung Department of Health (DOH) Hospital, the Taoyuan DOH Hospital, the Lishin Hospital and the Minsheng Hospital. Probands inclusion criteria are as follows: (1) systolic blood pressure (SBP) >140 mmHg and/or diastolic blood pressure (DBP) >90 mmHg or currently on antihypertensive medication for a minimum of one year; (2) age <40 years old at first diagnosis of hypertension; (3) secondary causes of hypertension (such as chronic renal disease, renal arterial stenosis, primary aldosteronism, coarctation of the aorta, thyroid disorders, Cushing syndrome, and pheochromocytoma) were excluded through extensive clinical examination and investigation (including blood chemistry, renal function tests, endocrine examination, and abdominal sonogram); (4) absence of insulin-dependent diabetes mellitus; and (5) body mass index (BMI) <30 kg/m^2^. A total of 305 probands met the criteria and agreed to participate in the study. The spouse and all first-, second- and third-degree relatives of each proband were also invited to join the study.

Data collection was carried out according to standardized protocols. Blood pressure, pulse, height and weight were measured according to the protocol established for the Nutrition and Health Survey in Taiwan [18]. Serum ACE activity (IU/l, nmol/min/ml) was measured on plasma prepared from fasting blood samples (10–12 hour fast) using the spectrophotometric method (ACEcolor, Fujirebio Inc.) [19]. The within-run and between-run coefficients of variation for this assay were 3.5% and 5.2%, respectively. In addition, data on socio-demographic factors, smoking, drinking, physical activity and other lifestyle habits, socio-demographic factors, past medical history and medications were collected via interviews.

### Research design of the main study

A two-stage design was used to fine-map the ACE activity QTLs that flanked the ACE structural gene located on chromosome 17q23. By using the Han Chinese SNP database with SNPbrowser software (Applied Biosystems), we selected 41 SNPs (distributed over *ACE* and another 11 genes) based on Assay-on-Demand (Applied Biosystems) with a minor allele frequency (MAF) of 0.2 or more (data not shown) within and around the *ACE* gene and within 1 cM of the highest two-point and multipoint LOD scores from the results of our previous study [17]. Association tests showed that only one (rs4353) out of six SNPs (rs4293, rs4295, rs4353, rs4575595, rs11868324, and rs4267385) located in the *ACE* gene was statistically significant after Bonferroni correction.

Following up the result of the stage I; in stage II, we focused on mapping within ACE gene, identifying the LD structure, showing relationships between tag-SNPs and ACE activity and hypertension. Twenty-five additional SNPs with a MAF greater than 10% (23.68% to 42.19%) were selected from the SNP database established by NCBI (dbSNP BUILD 126) including: rs8076157, rs4277405, rs4459609, rs1800764, rs4291, rs4292, rs4305, rs4309, rs4311, rs4316, rs4320, rs4324, rs4329, rs4331, rs4332, rs4335, I/D polymorphism, rs4342, rs4343, rs4344, rs4351, rs4359, rs4362, rs4363, and rs4366. Statistical analysis was carried out to localize QTLs with the genotypic information of these 25 SNPs and 6 previous ones among which there were 5 synonymous coding SNPs. Major haplotypes were constructed in the region most significantly associated with ACE activity in order to pinpoint variants affecting serum ACE activity. Furthermore, we measured total ACE mRNA level by using real-time PCR to reconfirm association found between haplotypes and ACE activity. Finally, replication studies were performed to confirm the associations between significant SNPs and ACE activity and hypertension.

### Replication studies

To confirm the association between ACE activity and *ACE* variants, ACE activity was measured for another 1023 young-onset hypertension patients from the Academia Sinica young-onset hypertension multi-center study [20], which adopted the same inclusion criteria as described above except for a different age of onset (<50 years). The institutional-review-board and informed-consent processes were handled as above.

We conducted second-stage replication to confirm the association between young hypertension and *ACE* variants by genotyping 421 young hypertension patients randomly selected from the above multi-center study and additional 421 age- and gender-matched controls from the Han Chinese Cell and Genome Bank in Taiwan [21]. Five major tag-SNPs were selected and genotyped to represent each of the four LD blocks.

### Genotype data

Genomic DNA was extracted using phenol-chloroform method from the buffy coat layer of the peripheral blood. In stage I, 41 SNP markers were performed using TaqMan Assays (Applied Biosystems) genotyped with ABI 7900 HT [22]. In stage II, a total of 25 SNPs except for I/D and rs4366 were carried out using matrix-assisted laser desorption ionization time-of-flight (MALDI-TOF) mass spectrometry (Sequenom) [23] and TaqMan assays. PCR was used to determine the allele type for the I/D polymorphism in intron 16 [24] and rs4366 in the 3′ UTR was genotyped using an ABI 3730 DNA analyzer and GeneMapper 3.0 software (Applied Biosystems).

### ACE expression study

The selection of subjects for mRNA expression experiments was made according to their ACE haplotypes. We randomly selected eight subjects from those with homozygous haplotype 1 and another eight subjects from those with homozygous haplotype 2. The cDNA was synthesized from RNA extracted from lymphocyte cells using the RNeasy Mini kit (Qiagen, Courtaboeuf, France), including the RNase-free DNase step to remove any trace of potentially contaminating genomic DNA. The ACE mRNA expression levels were measured by real-time PCR quantification and compared between two haplotypes. The mRNA expression values were normalized to ß-actin expression levels. The correctness of the product sizes were verified by gel electrophoresis.

### Statistical analysis

The SAS program (SAS Institute Inc.) was used to assess Mendelian inheritance and Hardy-Weinberg Equilibrium for all the markers. In stage I, family-based association (FBAT software) test [25] was used to examine whether the 41 markers flanking ACE and other nearby genes were significantly associated with the square root of ACE activity.

In stage II, we used Haploview (version 4.1) [26] to estimate the pairwise LD between the 31 SNPs on ACE, using the LD-block definition obtained from Gabriel et al [27]. Four LD blocks were constructed and their tag-SNPs were identified. A multivariate generalized linear model with a Gaussian link function was used to test the differences in mean ACE activity between genotypes constructed from tag-SNPs and from all other SNPs. The SAS haplotype procedure was used to determine haplotypes for all studied subjects. The effects of anti-hypertensive medications were adjusted as dummy variables in all analyses carried out.

Young-onset hypertension was treated as a binary trait, and was regressed on genotypes constructed from measured genotyped tag-SNPs using a generalized linear model with a logit link function, adjusting age, gender, and anti-hypertensive medications as covariates and adjusting family structure by using a generalized estimating equation (GEE) [28].

## Results

### Study subject characteristics

The average levels of ACE activity, the square root of ACE activity, BMI, SBP, DBP, and the proportions of cigarette smoking and alcohol consumption were significantly higher in males than in females (Table 1). There were 240 male and 164 female young-onset hypertension patients. Their average age of onset were 33 and 34, respectively. The most commonly prescribed anti-hypertensive medications were β-adrenergic blocking agents, followed by ACE inhibitors and then angiotensin receptor blockers for these patients. The effects of these medications on subject parameters were carefully adjusted in all the following analyses.

**Table 1:** Characteristics of the participants.

Characteristics	Men Mean ± SD	Women Mean ± SD
All participants (n)	597	571
Age at recruitment, y	46.2±14.9	48.2±14.4*
ACE activity, IU/l	15.0±6.9	13.7±5.7*
SqrtACE activity, (IU/l)^1/2^	3.8±0.8	3.6±0.7*
BMI, kg/m^2^	25.7±3.7	25.0±4.1*
SBP, mm Hg	131.6±20.1	129.3±21.5*
DBP, mm Hg	83.9±14.6	79.3±13.3*
Smokers, n (%)	323 (54.1)	31 (5.4)*
Alcohol consumption, n (%)	188 (31.5)	27 (4.7)*
Young-onset hypertensive patients (n)	240	164
Onset age, y	33.3±6.1	34.3±5.5*
Antihypertensive use, n (%)	112 (46.7)	97 (59.2)
β-adrenergic blocking agents	55 (22.9)	43 (26.2)
ACE inhibitors	27 (11.3)	31 (18.9)
Angiotensin receptor blockers	31 (12.9)	17 (10.4)

Young-onset hypertension is defined by SBP >140 mm Hg, DBP >90 mm Hg, or taking hypertensive medications and onset age ≤40 years.

SqrtACE activity, square root of angiotensin-converting enzyme activity;

BMI, body mass index; SBP, systolic blood pressure; DBP, diastolic blood pressure.

* p<0.05 for comparison between men and women, using the mixed model controlled for intra-family correlation.

### Fine-mapping

**Figure 1:**
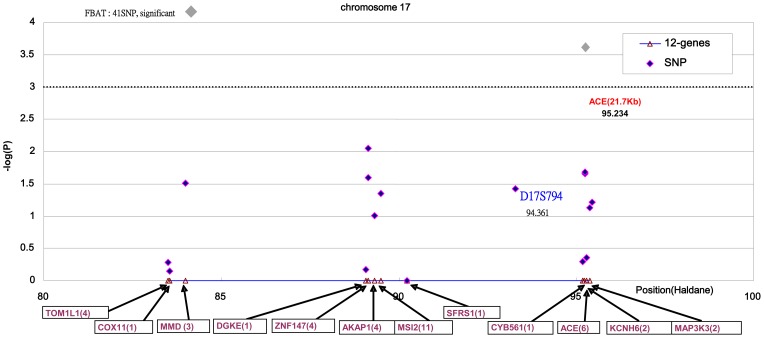
The first-stage fine-mapping for ACE activity QTL with 41 SNPs flanking ACE structural gene and the surrounding region on chromosome 17q23. We selected 41 SNPs (distributed over *ACE* and another 11 surrounding genes) based on Assay-on-Demand (Applied Biosystems) by using the Han Chinese SNP database with SNPbrowser software. The number of SNPs for each gene is shown in parentheses.

**Figure 2:**
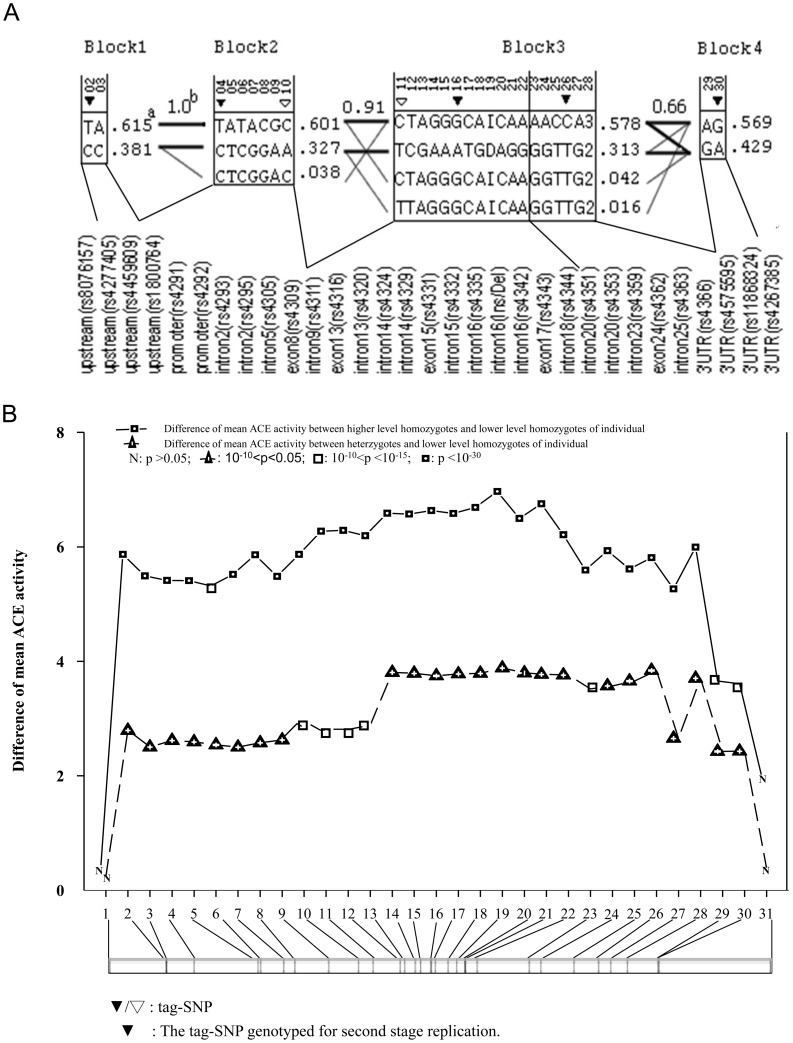
The second-stage fine-mapping for ACE activity QTL on the ACE structural gene. Haplotype, tag-SNP, and LD block information derived by Haploview and genotyped tag-SNPs for verification study are shown in the top figure. The rs number for each SNP is provided. The numbers at the top of the upper panel in Figure 2(A) and at the bottom of the lower panel in Figure 2(B) are the serial numbers of the 31 genotyped SNPs. The open and filled triangles in Figure 2(A) display tag-SNPs. The filled triangles are those selected for verification study. ^a^A value displays frequency of the haplotype in the LD block on the left side. ^b^A value of multiallelic D' representing the level of recombination between the two blocks. Figure 2(B). Differences of mean ACE activity between genotypes (solid and dashed lines) were determined individually for each SNP by generalized estimating equations (GEE), and adjusted for age, gender, and antihypertensive medications. The distribution and relative locations of the 31 SNPs are shown in the lower panel of Figure 2.

In stage I, only the SNP-rs4353 within *ACE* had a statistically significant association (−log_10_(p)  = 3.6>2.91) among the 41 SNPs flanking the *ACE* gene, after carrying out the Bonferroni correction (Figure 1). In stage II, the genotypic information of an additional 25 SNPs and 6 previous ones was used to localize variants affecting serum ACE activity. The haploview program identified four LD blocks from these 31 SNPs (the upper panel of Figure 2). The first LD block contains two SNPs in the upstream region (rs4277405 and rs4459609) spanning 30 base pairs (bp). The second LD block consists of seven SNPs (rs1800764 in the upstream region, rs4291 and rs4292 in promoter region, three intron SNPs, and rs4309 in exon 8) and spans 9.4 kb. The third LD block includes 17 SNPs (from rs4311 in intron 9 to rs4366 in the 3′ UTR region, including the I/D polymorphism on intron 16) and spans 14.7 kb. The fourth LD block contained two SNPs in the 3′UTR region (rs4575595 and rs11868324), which covers 94 bp. There are one, two, three and one tag-SNPs, respectively, in each of the LD blocks.

### Relationships between *ACE* genotypes, ACE activity, and young-onset hypertension

According to the above results, five major tag-SNPs were selected and genotyped to represent each of the four LD blocks (filled triangles in the upper panel of Figure 2). We found, in the family study (upper part of Table 2), that each tag-SNP was significantly associated with ACE activity (p-value ranges from 10^−16^ to 10^−33^) after adjusting for the family structure and for the covariates of age, gender, and antihypertensive medications in a generalized linear model with Gaussian link function. Among the five tag-SNPs, subjects having the AA and AG genotypes of rs4331 on exon 15 displayed the highest ACE activity levels compared to subjects having the GG genotype. Our replication study yielded similar results with 842 independent hypertensive subjects aged 20–50 from the AS multi-center study (lower part of Table 2).

**Table 2:** ACE activity and risk of acquiring young-onset hypertension among people with different genotypes.

Parameter			ACE activity (IU/l)		Young-onset hypertension	
LD block	Tag-SNP	n	Estimate (SE)	p-value	OR (95% CI)	p-value
Stage I: YOH hypertension family study*						
1	Rs4277405					
	CC	104	16.10 (0.74)	<1×10^−30^	1.08 (0.64,1.81)	0.7800
	CT	431	12.80 (0.59)	6.24×10^−10^	0.84 (0.60,1.17	0.3023
	vs TT	257	10.05 (0.70)		1	
2	rs1800764					
	CC	121	16.32 (0.69)	<1×10^−30^	1.50 (0.94,2.40)	0.0880
	CT	456	13.34 (0.56)	2.40×10^−9^	0.89 (0.65,1.21)	0.4505
	vs TT	412	10.70 (0.70)		1	
	CC	121			1.61 (1.03,2.51)	0.0358
	vs CT+TT	884			1	
3	rs4331					
	AA	90	16.77 (0.75)	<1×10^−30^	1.19 (0.69,2.03)	0.5287
	AG	428	13.68 (0.75)	1.03×10^–8^	0.75 (0.54,1.02)	0.0701
	vs GG	417	9.91 (0.59)		1	
	rs4362					
	TT	135	15.62 (0.74)	<1×10^−30^	0.90 (0.57,1.44)	0.6637
	CT	449	13.31 (0.75)	6.33×10^−10^	0.70 (0.51,0.96)	0.0275
	vs CC	346	9.29 (0.59)		1	
4	rs1186832					
	AA	182	14.81 (0.72)	4.47×10^−11^	0.76 (0.50,1.15)	0.1906
	AG	487	13.38 (0.73)	2.52×10^−4^	0.76 (0.55,1.07)	0.1194
	vs GG	334	10.88 (0.65)		1	
Stage II: multi-center YOH association study						
1	rs4277405					
	CC	82	17.76 (0.79)	7.9×10^−17^	0.87(0.55,1.38)	0.5459†
	CT	339	14.75 (0.26)		1.17 (0.74,1.85)	0.5028†
	vs TT	307	11.90 (0.37)		1	
2	rs1800764					
	CC	90	17.58 (0.73)	1.30×10^−16^	1.12 (0.72,1.74)	0.6276†
	CT	341	14.75 (0.26)		1.41 (1.05.1.89)	0.0216†
	vs TT	295	11.85 (0.38)		1	
	CC + CT	471			1.34 (1.02,1.77)	0.0380†
	vs TT	356			1	
3	rs4331					
	AA	73	19.24 (0.89)	2.34×10^−33^	1.18 (0.75,1.87)	0.4716†
	AG	306	15.42 (0.38)		1.21 (0.91,1.61)	0.1998†
	vs GG	334	11.20 (0.20)		1	
	rs4362					
	TT	109	18.42 (0.67)	1.19×10^−30^	1.16 (0.78,1.77)	0.4783†
	CT	333	14.83 (0.36)		1.12 (0.83,1.50)	0.4537†
	vs CC	287	11.10 (0.21)		1	

* The estimates and standard error (SE) of ACE activity, the odds ratio (OR) and the 95% confidence interval (CI) of young-onset hypertension were performed individually for each SNP by generalized estimating equations; adjusted for age, gender, and drugs; and controlled for intra-family correlation.

† Young-onset hypertension was treated as a binary trait, and was regressed on genotypes constructed from measured genotyped tag-SNPs using a generalized linear model with a logit link function, adjusting age, gender, and anti-hypertensive medications.

The odds ratios (OR) from a generalized linear model with a logistic function for associations between five tag-SNPs and young-onset hypertension for both family study and replication case-control study are also shown in Table 2. People with the CC genotype in rs1800764 (−3892 bp) had a greater risk (OR  = 1.61, p = 0.0358) of being a young-onset hypertension patient when compared with those with CT/TT genotypes. This SNP of the LD block 2 is in LD with two promoter SNPs and with SNPs in intron 2, intron 5, and exon 8 (the upper panel of Figure 2). However, the degree of association between hypertension and other tag-SNPs was much lower and not statistically significant and the magnitude of the association between the CC genotype in rs1800764 and hypertension was moderate compared to that with ACE activity. This association between young-onset hypertension and rs1800764 was reconfirmed in another 421 unrelated hypertension cases and 421 age- and gender-matched controls with an OR of 1.34 (p = 0.038), when comparing genotype CC/CT to TT.

### ACE mRNA expression in lymphocyte cells with two distinct haplotypes

We selected thirteen SNPs distributed throughout the ACE gene including 7 tag-SNPs and 6 SNPs in functional positions. First, we used SAS genetics with proc haplotype procedure to estimate haplotypes for thirteen SNPs (Table 3). The six most frequent haplotypes which cumulatively account for 93.2% of the observed haplotypes and the mean levels of each haplotype for ACE activity are shown in the Table 3. The two most frequent haplotypes, haplotype 1 (T-T-A-T-T-C-T-G-I-A-C-3-G) and haplotype 2 (C-C-T-C-C-T-C-A-D-G-T-2-A), complement each other in the yin-yang fashion at all 13 sites. The mean ACE activity levels of haplotype1 were much lower than those of haplotype2 (P value = 2.36×10^−30^).

**Table 3:** Ranked ACE haplotypes frequencies and means of serum ACE levels.

Haplotypes	rs4277405	rs1800764	rs4291	rs4292	rs4309	rs4311*	rs4316*	rs4331*	I/D*	rs4343*	rs4362*	rs4366*	rs11868324	N	Percent (%)	Percent (%)	Mean ± SD
1	T	T	A	T	T	C	T	G	I	A	C	3	G	994	48.49	48.49	12.88±4.91
2	C	C	T	C	C	T	C	A	D	G	T	2	A	507	24.73	24.73	16.56±5.26
3	T	T	A	T	T	C	T	G	I	A	C	3	A	189	9.22	9.22	13.13±4.89
4	C	C	T	C	C	T	C	A	D	G	T	2	G	85	4.15	4.15	16.61±5.51
5	T	T	A	T	T	C	T	G	I	A	T	2	A	74	3.61	3.61	13.79±3.96
6	C	C	T	C	T	C	T	G	I	A	C	3	G	62	3.02	3.02	13.16±5.34

* The bin from rs4311 to rs4366 for further fine-mapping.

For the bin from rs4311 to rs4366, subjects with T-C-A-D-G-T-2 haplotype had higher mean ACE activity levels than those with C-T-G-I-A-C-3, irrespective of the haplotype from rs4277405 to rs4309. To determine which variants on *ACE* gene influence ACE activity variation, we genotyped 4 additional SNPs (rs4324, rs4344, rs4353 and rs4359) between rs4311 and rs4366 for all samples and performed the same haplotype analysis (Table 4). A recombinant breakpoint was found between rs4344 and rs4353. Interestingly, the mean ACE activity among subjects with the G-T-T-2 haplotype estimated from SNP-rs4353 to SNP-rs4366 increased by 1.3 IU/l, compared to the A-C-C-3 haplotype. In another region from SNP-rs4316 to SNP-rs4344, the mean ACE activity among subjects with the C-A-A-D-G-G haplotype increased by 2.3 IU/l compared to the T-G-G-I-A-A haplotype. The haplotypes showed additive effects on ACE activity and increment was about 1.3–2.3 IU/l per copy of the haplotype.

**Table 4:** Additional SNPs between rs4311 and rs4366 to determine which variants influence ACE activity variation.

Haplotypes	rs4311*	rs4316*	rs4324†	rs4331*	I/D*	rs4343*	rs4344†	rs4353†	rs4359†	rs4362*	rs4366*	N	Percent (%)	Mean ± SD
A	T	C	A	A	D	G	G	G	T	T	2	642	31.32	16.57±5.27
B	C	T	G	G	I	A	A	G	T	T	2	107	5.21	14.38±5.11
C	T	T	G	G	I	A	A	G	T	T	2	30	1.46	14.07±3.91
D	C	T	G	G	I	A	A	A	C	C	3	1271	62	12.94±4.92

* SNPs in stage-1 haplotype analysis.

† Additional SNPs.

**Figure 3:**
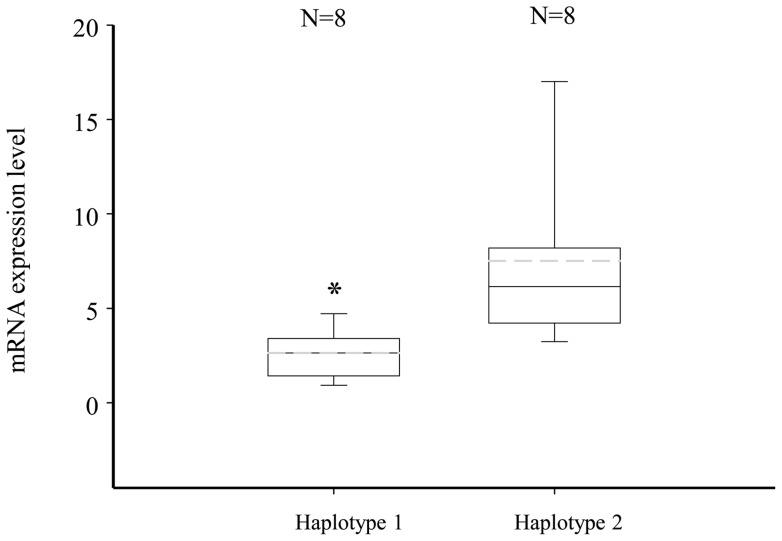
Total mRNA expression levels of angiotensin I–converting enzyme in lymphoblast cells. Box plots display mean expression ± one quartile. Gray medium dash line stands for mean level. Results are grouped by haplotype. ACE mRNA levels are relative to β-actin. * P<0.05 vs. Haplotype 2.

The two major haplotypes were found to be significantly associated with serum ACE activity in both family and replication studies. In order to confirm these results, mRNA levels of subjects homozygous for the two major haplotypes were quantified by real-time PCR. Haplotype1exhibited a significantly lower level of expression of ACE gene than haplotype 2 (p = 0.0032; Figure 3).

## Discussion

The *ACE* I/D polymorphism has been a focus of *ACE* genetic studies for some time. Although ACE activity has been shown to increase with the number of D alleles carried [9], it is unclear whether the I/D polymorphism is functional. We demonstrated that multiple and consecutive SNPs (including the I/D polymorphism) on the second and third LD blocks of *ACE*, as we denoted in this paper, were significantly and uniformly associated with variations in ACE activity (Figure 2). Thus, we constructed two major haplotypes spanning LD blocks 2 and 3. The mean ACE activity increased as the number of the ACE elevating haplotypes rose (Table 3). On the other hand, rs1800764, the tag-SNP representing the LD block 2 of *ACE*, was the only SNP that was moderately but significantly associated with young-onset hypertension. Our fine-mapping results for ACE activity and for young-onset hypertension were confirmed by additional sets of independent samples.

**Figure 4:**
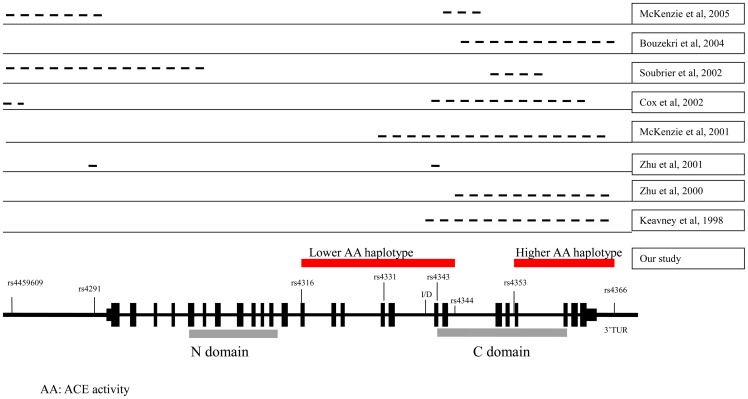
Potential SNPs or regions of the ACE gene influencing inter-individual variation of the circulating ACE activity in present and previous studies.

Figure 4 compares our findings with those of previous studies that searched for the location of *ACE* variants responsible for activity variations. The previous studies were carried out on various ethnic groups and adopted different methodologies, such as variance components analysis, direct sequencing, measured haplotype analysis, and use of cladistic methods [7], [11], [12], [13], [14], [15], [16]. Most studies found that a candidate region from exon15 to 3′UTR contains one or more variants in LD, with a functional variant affecting serum ACE activity (Figure 4). Our study selected 31 SNPs throughout *ACE* (roughly one per every 1.2 kb) and found a primary region from exon 13 (rs4316) to intron18 (rs4344), which includes the ID polymorphism responsible for decreased ACE activity, and a secondary region from intron 20 (rs4353) to 3′UTR (rs4366), which is responsible for elevated ACE activity by measured haplotype method. A recombinant break point between rs4344 and rs4353 appears to define both the G-T-T-2 haplotype (elevated ACE activity level) and the T-G-G-I-A-A haplotype (decreased ACE activity level). Although another recombinant break point was observed between rs4292 and rs4309, the bin between rs4277405 and rs4292 could not account for the variation in ACE activity. Additionally, our previous study that a variant (rs4343) at exon 17 in decreased ACE activity region was associated with response to ACEI [20]. We identified two functional haplotypes affecting plasma ACE levels and potential response to ACEI. If these two haplotypes can be substantiated by a clinical trial to examine BP response to the ACEI, it may help determine whether ACEI is the choice of antihypertensives for given individual patients.

Furthermore, we found that the magnitudes of the difference in mean ACE activity between subjects with two major haplotypes were similar to that between all SNP allele types spanning LD blocks 2 and 3. This indicates that a very long haplotype may be responsible for the ACE activity. Although our data showed that the total mRNA expression levels were associated with haplotypes, further functional studies are required to pinpoint exactly where the ACE activity QTL is located.

Human somatic ACE is a type I integral membrane glycoprotein [29]. Its extracellular segment consists of two domains, the N-terminal (from exon 4 to exon 11) and C-terminal domain (from exon 17 to exon 24), each containing an M2-type (peptidyl-dipeptidase) zinc metallopeptidase motif – HEXXH [30]. The specific activity of the C-terminal domain is dependent on chloride concentration, which is critical for ACE activity [31]. Corvol et al. [29] showed that the N-terminal domain may have less to do with the RAS. The differences in the two domain functions may in part explain why tag-SNPs in LD block 3 have slightly higher association with ACE activity and tag-SNPs in LD block 2 have higher association with young-onset hypertension. The I/D polymorphism in intron 16 is situated between the two metallopeptidase domains and in the middle of LD block 3 and thus may be considered as a surrogate of LD block 3.

With regard to young-onset hypertension, a moderate association was found with rs1800764, the tag-SNP of LD block 2 that was in strong linkage disequilibrium with SNPs in the *ACE* promoter, intron 2, intron 5, and exon 8 (the upper panel of Figure 2). This phenomenon was reconfirmed with an independent set of 421 hypertension-control pairs in our study. The etiology of hypertension is complex, and the RAAS pathway, which is not the only one involved, has multiple genes. For example, the genetic effect of angiotensinogen has been confirmed at a very moderate level by meta-analysis [32]. It is therefore not surprising that *ACE* variants have only a moderate effect on hypertension. Animal studies have indicated that N-terminal domain function of ACE is related to kidney development [29]. Further research is needed to examine whether the association between rs1800764 and hypertension occurs via elevated ACE activity or via another kidney mechanism. Because we have identified functional *ACE* polymorphisms for hypertension in regions several kb upstream of the I/D polymorphism, this may in part explain why consistent associations between I/D polymorphism and blood pressure/hypertension are lacking in the literature [33].

Regulation of ACE activity may be even more complex than the present and previous studies would suggest (Figure 2). Our results from the Asian population identified yet more potential *ACE* QTL on *ACE*. Although this *ACE* QTL mapping study was the first attempt of its kind in an Asian population, the original design was for mapping hypertension genes. Nonetheless, by simultaneous examination of the inter-relations between ACE activity, hypertension, and ACE variants, our data provide important insights into the genetic factors contributing to hypertension with relevance to East Asian populations and provides comparative data for Caucasian findings.

## Conclusions

In conclusion, we have discovered a moderate effect variant upstream of ACE promoter for young-onset hypertension and two *QTL*s of ACE activity, one from exon 13 to intron 18 and the other from intron 20 to 3′UTR. Their effects are additive.
